# Optical Simulation and Experimental Assessment with Time–Walk Correction of TOF–PET Detectors with Multi-Ended Readouts †

**DOI:** 10.3390/s21144681

**Published:** 2021-07-08

**Authors:** Siwei Xie, Zhiliang Zhu, Xi Zhang, Qiangqiang Xie, Hongsen Yu, Yibin Zhang, Jianfeng Xu, Qiyu Peng

**Affiliations:** 1 Institute of Biomedical Engineering, Shenzhen Bay Laboratory, Shenzhen 518107, China; xiesw@szbl.ac.cn (S.X.); zhuzl@szbl.ac.cn (Z.Z.); zhangyb@szbl.ac.cn (Y.Z.); 2 State Key Lab of Digital Manufacturing Equipment & Technology, School of Mechanical Science and Engineering, Huazhong University of Science and Technology, Wuhan 430070, China; xizhang@hust.edu.cn (X.Z.); xieqiang@hust.edu.cn (Q.X.); m201970550@hust.edu.cn (H.Y.)

**Keywords:** positron emission tomography, time of flight, coincidence timing resolution, dual-ended readout, optical simulation

## Abstract

As a commonly used solution, the multi-ended readout can measure the depth-of-interaction (DOI) for positron emission tomography (PET) detectors. In the present study, the effects of the multi-ended readout design were investigated using the leading-edge discriminator (LED) triggers on the timing performance of time-of-flight (TOF) PET detectors. At the very first, the photon transmission model of the four detectors, namely, single-ended readout, dual-ended readout, side dual-ended readout, and triple-ended readout, was established in Tracepro. The optical simulation revealed that the light output of the multi-ended readout was higher. Meanwhile, the readout circuit could be triggered earlier. Especially, in the triple-ended readout, the light output at 0.5 ns was observed to be nearly twice that of the single-ended readout after the first scintillating photon was generated. Subsequently, a reference detector was applied to test the multi-ended readout detectors that were constructed from a 6 × 6 × 25 mm^3^ LYSO crystal. Each module is composed of a crystal coupled with multiple SiPMs. Accordingly, its timing performance was improved by approximately 10% after the compensation of fourth-order polynomial fitting. Finally, the compensated full-width-at-half-maximum (FWHM) coincidence timing resolutions (CTR) of the dual-ended readout, side dual-ended readout, and triple-ended readout were 216.9 ps, 231.0 ps, and 203.6 ps, respectively.

## 1. Introduction

The effective axial length of current human Positron emission tomography (PET) systems is generally 25 cm to 35 cm, resulting in a number of effective events that are unable to be detected [1,2]. Therefore, the system’s sensitivity and image signal-to-noise (STN) ratio are relatively low. Increasing the axial length is an effective method in improving its sensitivity. Time-of-flight (TOF) was proposed by T. F. Budinger in 1977 [3], where positron annihilation positions were calculated by measuring the triggering time difference of two gamma photons. Therefore, by improving the timing performance of the detector while reducing the uncertainty of positron annihilation localization, the sensitivity of the PET system may be effectively improved [4,5,6,7].

The coincidence timing resolution (CTR) of the PET detector is influenced by various factors such as scintillator properties, structure, size, surface roughness, rise time, decay time, photosensor device, readout circuit, and threshold voltage [8,9,10,11,12,13]. Much research has been carried out on detector structure, timing measurement, and compensation algorithms [11,14,15,16,17,18,19,20,21]. J. Du et al. adopted a time–walk correction method. Timing resolution obtained using this method was equivalent to that obtained using a reference detector and was better than that obtained using constant fraction discriminators [16]. In regard to PET detectors based on discrete scintillators with a single-ended readout, scintillator size directly affects the TOF and trajectory of scintillating photons in the scintillator. S. Gundacker et al. used a one-to-one coupling structure to study the effects of various parameters including scintillator size, SiPM bias voltage, and trigger threshold on-time resolution. Accordingly, they found that the smaller the scintillator size is, the better the timing performance is [22]. Sandra Oliver et al. designed a dedicated cardiac TOF–PET system [23]. Although timing performance would increase with a decrease in scintillator length, too short of a scintillator structure would be unable to block additional gamma photons, reducing the detection efficiency and sensitivity of the TOF–PET system.

In terms of PET detectors with a dual-ended readout, the interaction depth measurement of γ photons in the scintillator helps improve the spatial resolution as well as correct the time information [24,25,26]. The dual-ended readout shortens the flying distance and trigger time of the scintillating photons to the photoelectric device while improving the timing performance of the detector. S. Seifert et al. adopted a 3 × 3 × 20 mm^3^ LSO scintillator with a dual-ended readout, in which its triggering time was corrected by the depth-of-interaction (DOI) information. The etching and polished scintillator obtained an FWHM CTR of 180 ps and 174 ps, respectively [26]. Time-to-digital converter (TDC) measurements are affected by baseline drifts that are mainly caused by past events and random noises from trigger timing (photon statistics, voltage settings, electronic noises, etc.) [27]. The advantage of having a multi-SiPM readout is that it reduces the effects pertaining to random noises and baseline drifts by sharing lights between multiple SiPMs. Cates J.W. et al. studied the side-ended readout detector and achieved a good time resolution [28,29].

Leading-edge discriminator (LED) triggers are often applied in TOF detectors employing numerous small SiPMs as they require fewer components in the analog implementations and may be set to a low level to trigger the initial scintillating photons [30]. The disadvantage of LEDs is that the timing measurement is affected by the signal amplitude; when two simultaneous pulses have the same shape, the larger the amplitude of the pulse, the earlier the trigger time. Therefore, an energy-based time compensation is necessary for LED triggers in order to effectively improve the timing performance of the TOF–PET detector [31].

In this paper, the timing performance of three multi-ended readout PET detectors was tested, analyzed, and compared. Combined with a photon transmission model and optical simulation via Tracepro [32], a polynomial fitting compensation algorithm was proposed. Compared with GATE, this software has a simple modeling method and faster simulation speed. After the use of the calibration algorithm of time–walk by energy for multi-terminal readout detector when LED is triggered, the time resolution of the detector has been improved to a certain extent.

## 2. Materials and Methods

### 2.1. Optical Simulation

The light output of the Lu_1.8_Y_0.2_SiO_5_ (LYSO) scintillator is about 26,000 photons/MeV, and the refractive index is 1.82 [33]. γ photons with an energy of 511 keV can produce about 13,000 scintillating photons through interactions in the LYSO scintillator. After multiple reflections on the internal surfaces of the scintillator, the photons are generated as a result of photoelectric or Compton interactions between the 511 keV gamma and the scintillator and detected by silicon photomultiplier (SiPM) with a reaction area of 6 × 6 mm^2^. Scintillating photons were emitted from the interaction position at random angles. The distribution of many scintillating photons is similar to a uniform sphere. For a traditional single-ended readout, half of the photons fly toward the SiPM while the remaining half fly away from the SiPM. The first batch of scintillating photons that arrive at the SiPM triggers the readout circuit.

The Tracepro software combines Monte Carlo ray tracing and simulation analyses to describe the surface characteristics of optical materials including absorption, reflection, transmission, and scattering [34]. In this study, a photon transmission model for both single-end and multi-end readout detectors was initially established using Tracepro to observe the influence of the scintillating photon transmission trajectory on the detector’s light output and timing performance.

**Figure 1:**
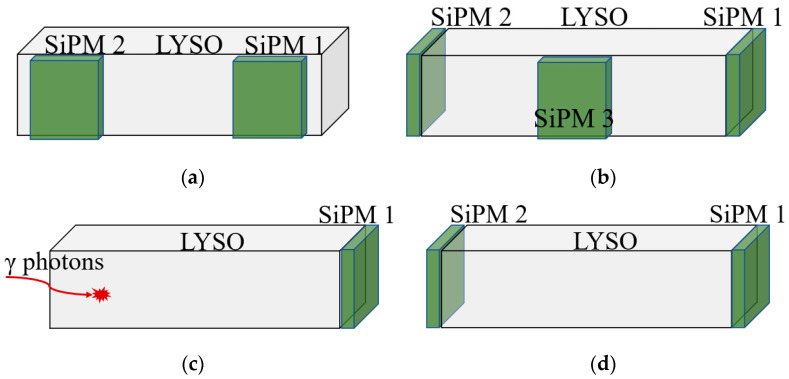
Detector models: (**a**) single-ended readout, (**b**) dual-ended readout, (**c**) side dual-ended readout, and (**d**) triple-ended readout.

As shown in Figure 1, four detector models based on the LYSO scintillator and SiPM were established including a single-ended readout (Figure 1a), dual-ended readout (Figure 1b), side dual-ended readout (Figure 1c), and triple-ended readout (Figure 1d). In the simulation, the size of the LYSO scintillator was 6 × 6 × 25 mm^3^, while the detection area of the SiPM was 6 × 6 mm^2^. The physical parameters of SiPM are set by referring to NUV SiPM (AdvanSiD). LYSO and SiPM were coupled using optical glue with a refractive index of 1.5. The side dual-ended readout detector, shown in Figure 1c, had two SiPMs coupled to the side surface of the scintillator. In regard to the triple-ended readout detector shown in Figure 1d, two SiPMs were coupled to the end of the scintillator, while one SiPM was coupled to the center of the side surface. The parameters (refractive index, absorption rate, thickness, and size) of the materials in the simulation are shown in Table 1.

**Figure 2:**
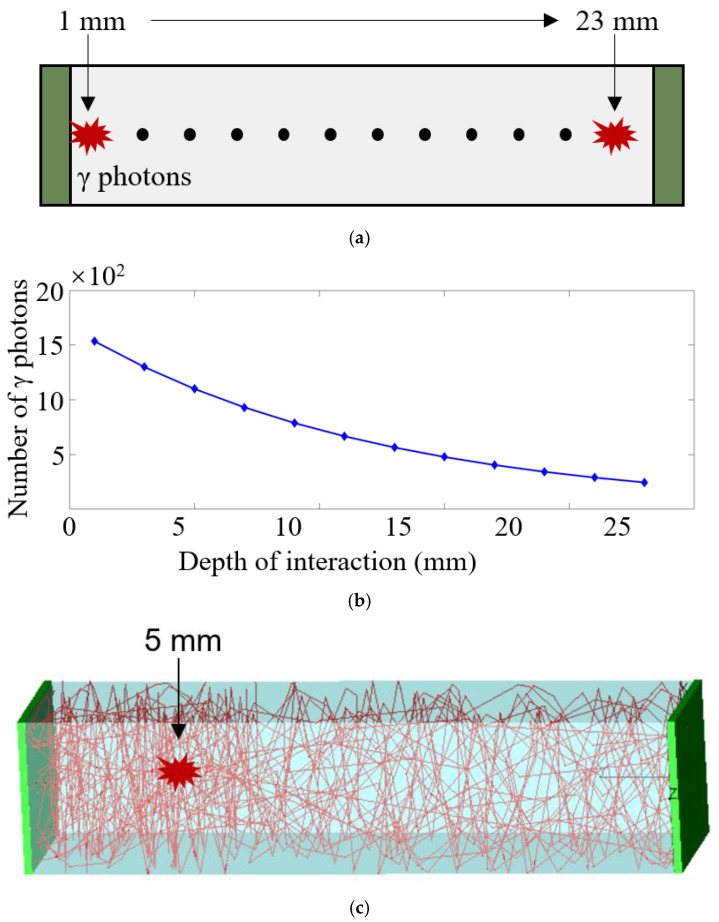
(**a**) The discrete interaction depth in the scintillator, (**b**) the number of γ photons generated at different depths, and (**c**) simulation model at the depth of 5 mm (only a few scintillating photons are shown in the picture).

The law of energy attenuation of γ-rays in the scintillators was found to be not linear. According to the Beer–Lambert law, the intensity of γ rays in the scintillator changes exponentially. Tracepro cannot directly simulate the attenuation distribution of γ photons within the scintillator. Therefore, the discrete position was used, as shown in Figure 2a, to simulate the energy distribution of gamma rays at different depths of the scintillator. At different depths, a group of scintillating photons possessing random flight directions was generated, where the number of scintillating photons was observed to change exponentially, as shown in Figure 2b. The initial number of incoming γ photons was 100 k. The attenuation length provided by the vendor of LYSO crystal was set to 12 mm for 511 keV. At a depth of 1 mm (0 mm to 2 mm), about 15.4 k γ photons were generated, while at a depth of 23 mm (22 mm to 24 mm), approximately 2.5 k γ photons were generated. Figure 2c shows the photon transmission model at the depth of 5 mm of a dual-ended readout detector. The trajectory (the red lines) of all scintillating photons in the scintillator was tracked until it was detected by the SiPM or absorbed by the scintillator. The energy and timing information of the scintillating photons were also recorded.

### 2.2. Multi-Ended Readout Detector

A reference detector was used to experimentally evaluate the timing performance of the multi-ended readout detector. The reference detector consists of a polished 2 × 2 × 2 mm^3^ LYSO scintillator and a NUV SiPM (AdvanSiD) [35] that has a detection area of 6 × 6 mm^2^. The uncoupling surface of the scintillator was wrapped with more than three layers of Teflon tape and coupled to the center of the SiPM with MeltMount glue.

**Figure 3:**
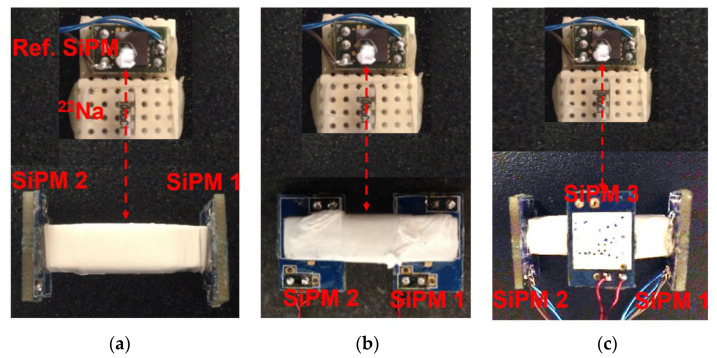
Coincidence experiment, (**a**) dual-ended readout, (**b**) side dual-ended readout, and (**c**) triple-ended readout.

Figure 3a–c show schematic diagrams of coincidence event acquisition for the dual-ended readout, side dual-ended readout, and triple-ended readout detectors in turn. The 6 mm NUV SiPMs were coupled to the end or side surfaces of the LYSO scintillator with MeltMount. Similarly, the surfaces of the scintillator that were not coupled with the SiPM were polished and covered with more than three layers of Teflon tape. A ^22^Na point source with an activity of 0.74 MBq was located between the reference detector and the test detector. According to previous research [36], the bias voltage of the SiPMs was set to 37.5 V to obtain better timing performance. All experiments were carried out in a light, tight box.

### 2.3. Readout Electronic Circuit

**Figure 4:**
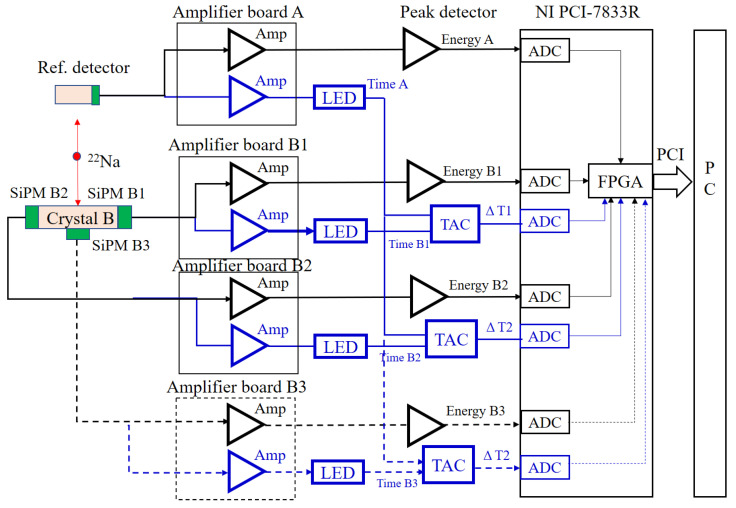
The diagram of the readout electronics system.

In the present study, an experimental system possessing excellent timing performance was constructed [10,27]. As shown in Figure 4, the two PET detectors were a detector with a multi-ended readout and a conventional reference detector.

Each custom-designed amplifier board split the signal from the SiPM into an energy signal and a timing signal that were then amplified. Two stages of current feedback amplifiers (AD8000) were chosen to amplify the timing signals with a total gain of 200. The −3 dB bandwidth and the slew rate of the amplifier were 1.5 GHz and 4100 V/μs, respectively. The amplified timing signals were fed to two LEDs (Model 454). With four channels of conventional constant fractional discriminators (CFDs), Model 454 is the device. Here, its internal circuits were modified, and the four CFDs were converted into LEDs as well. The outputs of the LEDs were fed to the time amplitude converter (TAC) that measured the timing interval between pulses to its start and stop inputs and generated an analog output pulse proportional to the measured time. The full scale of TAC was set to 50 ns, and, according to the datasheet, the FWHM of the timing resolution of Model 566 TAC was 10 ps for all ranges.

The outputs of the two TACs and the three amplified energy signals were connected to the NI PCI-7833R board that was a PCI-based reconfigurable I/O device with an FPGA and eight analog-to-digital converters (ADC) on-board. A LabVIEW program was developed to control FPGA to perform event triggering and read out energy and timing signals. The timing resolution of the readout electronics was calibrated with pulses that were generated from a signal generator.

Four energy signals were measured, including one signal from the reference detector ($E_{reference}$) along with two or three signals measured by SiPM 1 ($E_{1}$), SiPM 2 ($E_{2}$) or SiPM 3 ($E_{3}$), from the detector with a multi-ended readout. The time differences between the signal from the reference detector ($T_{reference}$) and the two or three signals ($T_{1}$, $T_{2}$, $T_{3}$) from the detector with a multi-ended readout were then measured. ∆T or ∆E represents the time or energy difference between the reference detector and multi-ended readout detector as follows:

(1) $$∆T=|T_{reference}-T|$$

(2) $$∆E=E_{reference}-E\;$$

### 2.4. Time–Walk Correction

**Figure 5:**
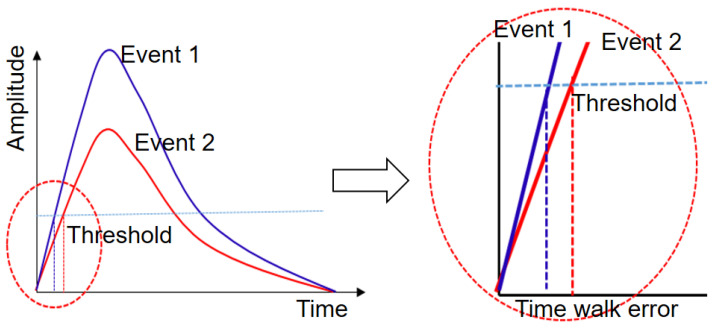
Schematic diagram of the time–walk for the LED trigger.

As shown in Figure 5, when the LED is used and a certain trigger threshold is set, if the waveform of the two pulses and the departure time are equal, the larger pulse will first reach the threshold. Correspondingly, when the energy of the two photons in the coincidence event varies, the trigger time also differs, reducing the time performance of the detector. The maximum output of the timing amplifier circuit was about 3 V. The mean amplitude of the waves of 511 keV events was approximately 2 V for both LYSO detectors; thus, the trigger level was set to 50 mV, which is approximately 2.5% of the amplitude of the 511 keV waves [31]. We proposed a fourth-order polynomial fitting method based on energy to compensate for the time difference generated by the LED trigger.

First, the energy and time signals of the coincidence events were counted, and the time and energy differences were calculated. Subsequently, the average measured time differences were computed, and the relationship between the average time difference $∆T_{m}$ ($∆T_{m1}$, $∆T_{m2}$, $∆T_{m3}$) and energy difference ∆E ($∆E_{1}$, $∆E_{2}$, $∆E_{3}$) was obtained. Finally, fourth-order polynomial fitting was carried out via MATLAB’s toolbox, and the fitted function f(∆E) was obtained. The compensated time difference $∆T_{c}$ was calculated by the measured time difference and the fitting function as follows:

(3) $$f\left(∆E\right)=fitting\left(∆E,\;mean\left(∆T_{m}\right)\right)$$

(4) $$∆T_{c}=\left|∆T_{m}-f\left(∆E\right)\right|\;$$

## 3. Results

### 3.1. Cumulative Density Light Output

**Figure 6:**
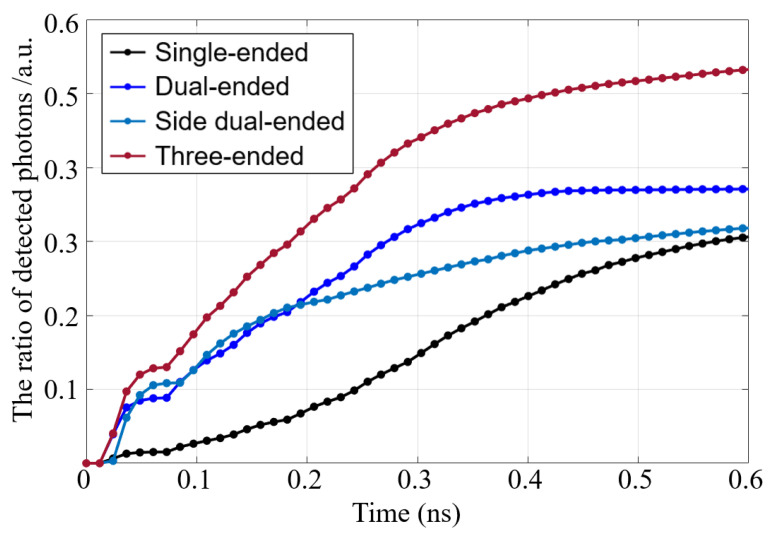
CDF of the light output of the simulation.

The cumulative distribution function (CDF) of the multi-ended readout detector was obtained by weighing the scintillating photons generated at different depths according to the probability distribution of γ-ray attenuation in the LYSO. Figure 6 shows the CDF curve depicting the number of scintillating photons detected by all SiPMs in the detectors. The X-axis represents the flight time of the scintillating photons, and the origin of the X-axis designates the time at which the scintillating photon was produced. The length of the flight path ($L_{flight}$) of the detected scintillating photon can be calculated by the following:

(5) $$L_{flight}=\frac{T_{flight}\times c}{n_{LYSO}}$$

where $T_{flight}$ is the flight time of scintillating photons, c is the speed of light (3 × 10^8^ m/s), and $n_{LYSO}$ is the refractive index of the LYSO scintillator. The end of the X-axis was set to 0.6 ns, signifying that the longest flight path of the detected scintillating photon is about 98.9 mm.

The corresponding results indicate that the photon transmission efficiency and light output of the multi-ended readout detector were improved to the single-end readout detector. To this effect, the light output of the triple-ended readout detector was observed to perform the best. When the flight time was 0.5 ns, the light output of the dual-ended, side dual-ended, and triple-ended readouts increase by 12.1%, 34.8%, and 85.9%, respectively, compared to the single-ended readout. Assuming that the trigger threshold was set to 1000 photons, the trigger time of the dual-ended, side dual-ended, and triple-ended readouts were about 1.51 ns, 1.56 ns, and 1.6 ns earlier than the single-end readout, respectively. Obviously, promptly improving the detector’s light output and photon transmission efficiency along with the scintillating photons reaching the SiPM possible can improve the detector’s timing resolution.

It should be noted that the interaction position of the discrete γ photons almost traverses the entire crystal in the simulation. The radioactive source was set at the direction of the depth (z = 0 mm). For a single γ photon, the light output and trigger time depend on the interaction position.

### 3.2. Energy Resolution

**Figure 7:**
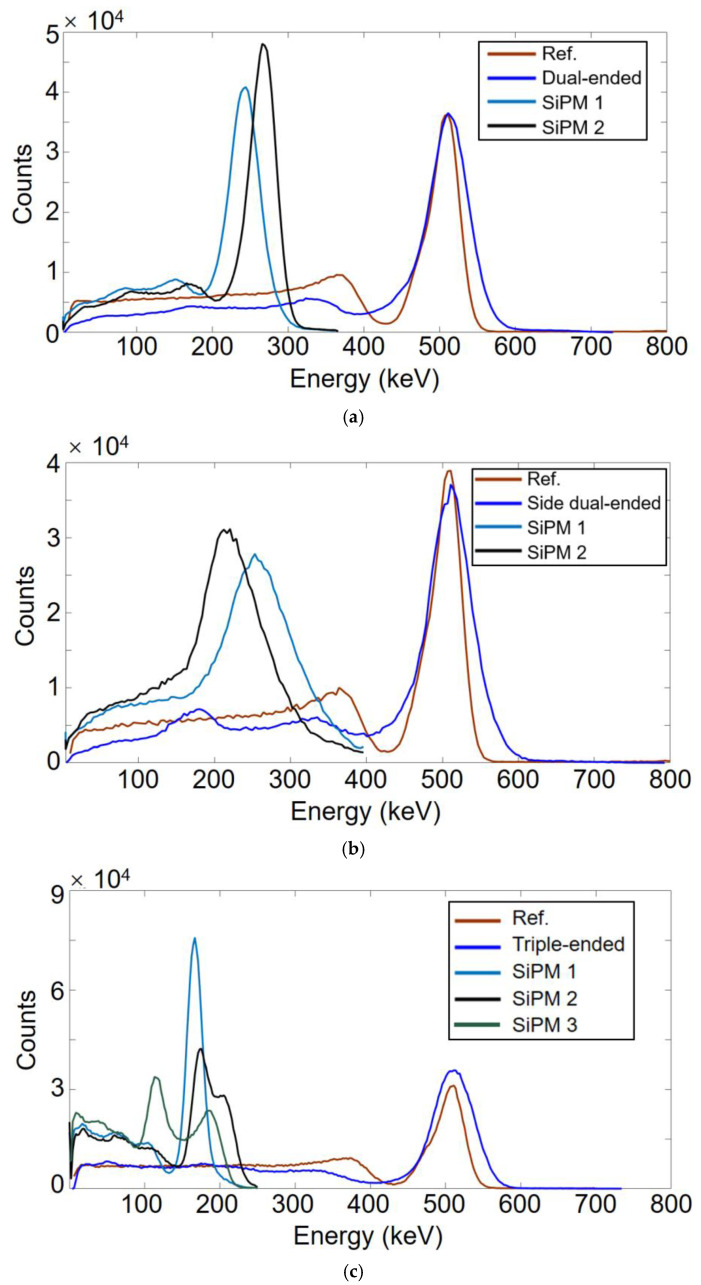
Energy spectra of (**a**) dual-ended, (**b**) side dual-ended, and (**c**) triple-ended readout experiment.

Figure 7a–c illustrates the energy spectra of the dual-ended, side dual-ended, and triple-ended readout experiments, respectively, including the energy spectra of SiPM1, SiPM2, and SiPM3 as well as the total energy for the test detector and reference detector. The peaks of the energy spectra for the reference detector and total energy of the multi-ended readout detector were normalized to 511 keV in a certain ratio to facilitate subsequent data processing.

According to the energy resolution of the detector, different energy windows were set in order to select valid events for the subsequent time–walk correction and timing resolution calculation. Table 2 outlines the energy resolutions (ER) and energy windows (EW) of the corresponding detectors. The energy resolution of the reference detector was similar in the three experiments, and the energy window of the reference detector was set to [440 550]. In addition, in regard to the multi-end readout test detectors, different energy windows were applied for SiPM 1, SiPM 2, and SiPM 3, respectively, excluding total energy.

### 3.3. Time–Walk Correction

**Figure 8:**
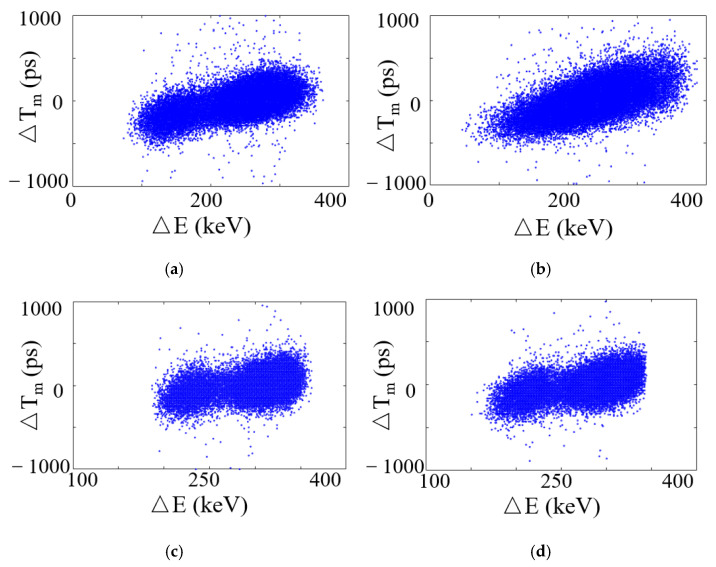
Scatterplots of $∆T_{m}$ and ∆E of (**a**) SiPM 1 of dual-ended readout, (**b**) SiPM 1 of side dual-ended readout, (**c**) SiPM 1, and (**d**) SiPM 3 of triple-ended readout detector.

Figure 8a–d depicts the scatterplots of the time difference ($∆T_{m}$) and energy difference (∆E) obtained by SiPM 1 in the dual-ended readout detector, SiPM 1 in the side dual-ended readout detector, as well as SiPM 1 and SiPM 3 in the triple-ended readout detector, respectively. It should be noted that the energy and time difference obtained by SiPM 2 is similar to that of SiPM 1; hence, it was not included in the figures.

**Figure 9:**
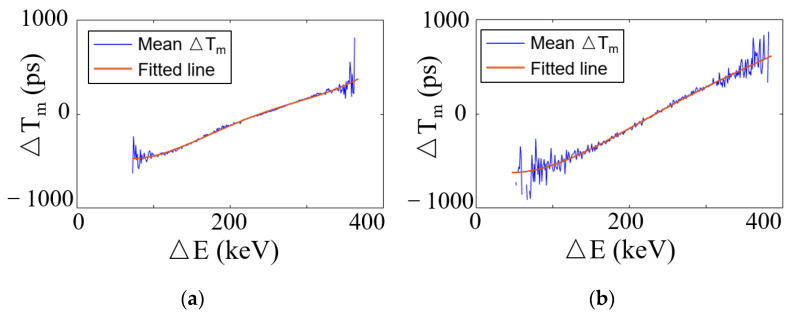

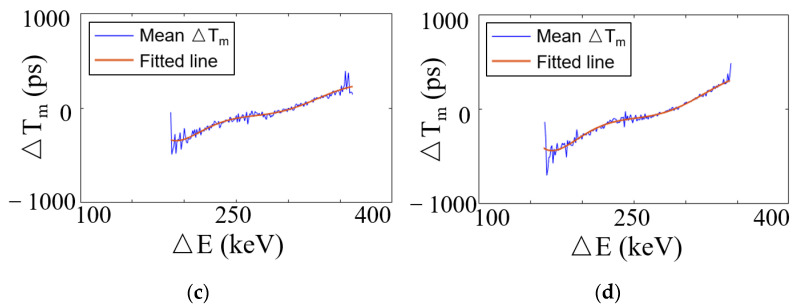
Relationships and fitted curve of mean $∆T_{m}$ and ∆E of (**a**) SiPM 1 of dual-ended readout, (**b**) SiPM 1 of side dual-ended readout, (**c**) SiPM 1, and (**d**) SiPM 3 of triple-ended readout.

Figure 9a–d shows the relationships and fitted curves of the energy difference (∆E) and average timing difference of Figure 8a–d. Notably, the time and energy differences do not represent simple linear relationships. Therefore, the fitted curve f(∆E) was obtained by fourth-order polynomial fitting via MATLAB’s toolbox. Both detectors show the same pattern, that is, as the energy difference increases, the time difference also gradually increases. Accordingly, it can be inferred that the larger the change in time difference is, the better the effect of time compensation is.

### 3.4. Timing Resolution

**Figure 10:**
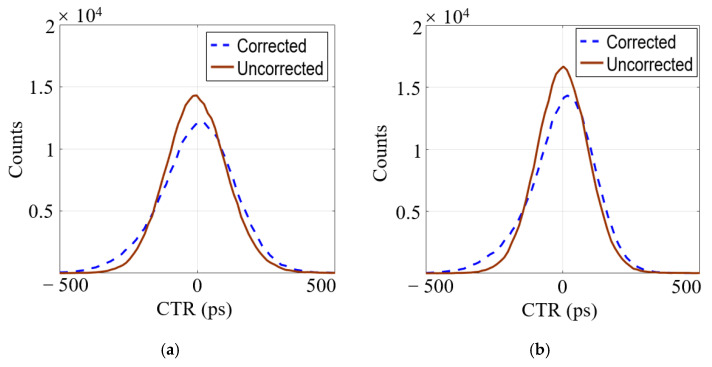

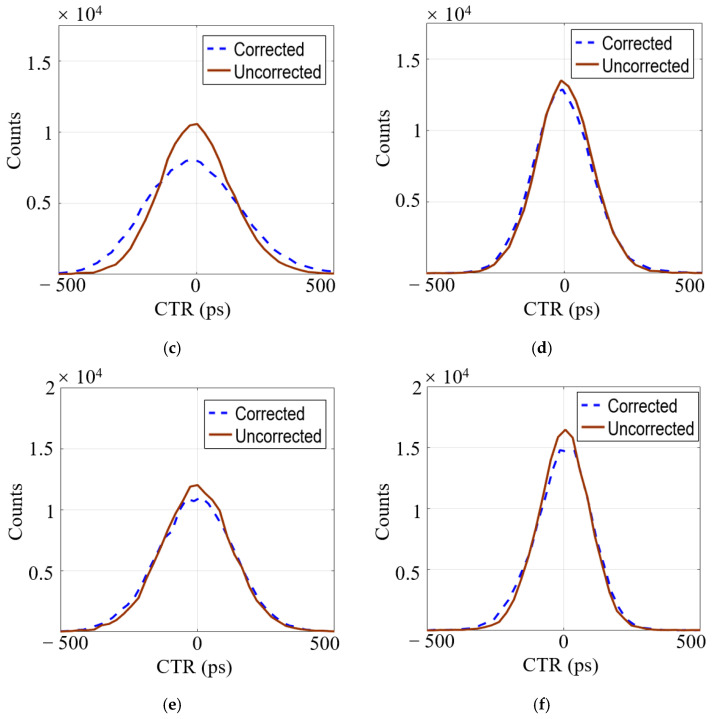
Time spectrum of (**a**) $∆T_{1}$ and (**b**) $∆T_{12}$ of the dual-ended readout; (**c**) $∆T_{1}$ and (**d**) $∆T_{12}$ of the side dual-ended readout; (**e**) $∆T_{1}$ and (**f**) $∆T_{123}$ of the triple-ended readout.

According to the measured time difference ($∆T_{m}$) and compensated time difference ($∆T_{c}$) calculated in the previous chapter, the time resolution of the multi-ended readout detector was computed. Figure 10 shows the compensated and uncompensated time spectrum obtained using SiPM 1 ($∆T_{1}$, Figure 10a) or using both SiPM 1 and SiPM 2 ($∆T_{12}$, Figure 10b) time signals in the dual-ended readout experiment, using SiPM 1 ($∆T_{1}$, Figure 10b) or using both SiPM 1 and SiPM 2 ($∆T_{12}$, Figure 10d) time signals in the side dual-ended readout experiment, and using SiPM 1 ($∆T_{1}$, Figure 10e) or using SiPM 1, SiPM 2, and SiPM 3 ($∆T_{123}$, Figure 10f) time signals in the triple-ended readout experiment. The figure demonstrates that the detector’s timing performance was improved after fourth-order polynomial fitting compensation. Compensated time difference using both SiPM 1 and SiPM 2 ($∆T_{c12})$, or using SiPM 1, SiPM 2 and SiPM 3 ($∆T_{c123})$ can be calculated by the following:

(6) $$∆T_{c12}=\left(∆T_{c1}+∆T_{c2}\right)/2$$

(7) $$∆T_{c123\;}=\left(∆T_{c1}+∆T_{c2}+∆T_{c3}\right)/3$$

**Figure 11:**
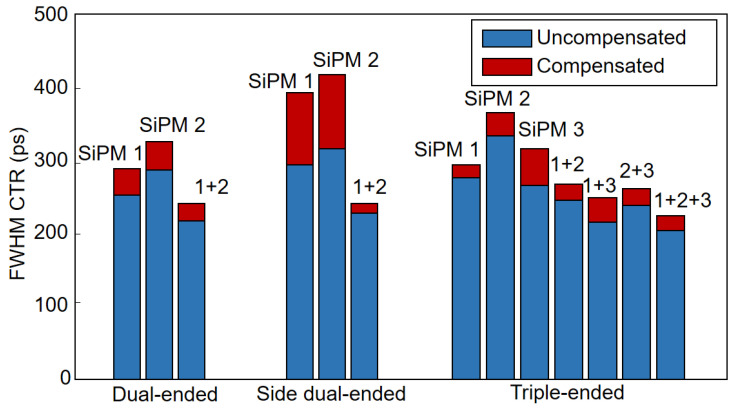
The FWHM CTRs of the tested detector.

The full-width-at-half-maximum (FWHM) CTR was used to comprehensively evaluate the timing performance for the multi-ended readout detectors. Table 3 shows the compensated and uncompensated CTR values as well as the promotion ratios of the three tested detectors. The promotion ratio was calculated according to the following formula:

(8) $$Ratio=\frac{CTR_{m}-CTR_{c}}{CTR_{c}}\times 100\%$$

where $CTR_{m}$ is the measured FWHM CTR value without compensation, and $CTR_{c}$ is the compensated FWHM CTR value. When all SiPM signals were used, the best CTR obtained by the dual-ended readout, side dual-ended readout, and triple-ended readout were 216.9 ps, 231.0 ps, and 203.6 ps, respectively. The performance improvement of the fourth-order polynomial fitting compensation algorithm was about 10%, and the maximum improvement was 25.2%, which appears in the side dual-end readout when only SiPM 1 is used. Figure 11 gives histograms of all measured FWHM CTRs. In the triple-ended readout experiment, the time signals of two SiPMs ($∆T_{12}$, $∆T_{13}$, $∆T_{23}$) were also utilized, and the resulting CTRs were 244.8 ps, 215.6 ps, and 238.6 ps, respectively, which was observed to be better than using one SiPM but worse than using three SiPMs.

## 4. Discussion and Conclusions

In this investigation, the size of the scintillator tested in the experiment was found to be relatively large; therefore, the FWHM CTR value was not less than 200 ps. According to previous research [37,38], due to the difference in the photon transmission path, the smaller the size of the scintillator is, the better the timing performance is. The CTRs shown in Table 3 were calculated by simply averaging the CTR measurements from two or three SiPMs. Calibration of the energy measurements is still underway, which may weigh the CTRs from the three SiPMs, further improving the timing performance of the PET detector.

The DOI decoding performance of the multi-ended readout was not analyzed in this study [39]. The scintillator surface was polished and coated with a high reflectivity Teflon tape. Therefore, the time–walk compensation only improves the timing performance by approximately 10%. Small-aperture animal PET systems with high spatial resolutions often use a dual-ended readout detector to obtain good DOI decoding performance. Here, small scintillators were covered with reflective materials that had low reflectivity, resulting in a decrease in energy resolution of the detectors. A larger energy window is needed to obtain a higher sensitivity, which may improve the effects of time–walk compensation based on energy.

The fourth-order polynomial was used for curve fitting, though it is not necessarily the best fitting method. Compared to other methods such as exponential fitting, logarithmic fitting, trigonometric functions, and higher-order polynomials, this method may obtain better time results.

The multi-ended readout detectors are helpful for the improvement of time performance of PET detectors, but they also have the following limitations: (1) The paper simply provides three ways of multi-channel coupling and examines their time performance. However, the triple-ended readout detector is difficult to build a clinical PET system with high sensitivity and spatial resolution because SiPMs are coupled to the side of the LYSO crystal, resulting in a decrease in the filling rate of the scintillator; (2) the reference detector with a small crystal size was used to evaluate the multi-ended readout detector in the paper. The results do not represent the time performance of a clinical PET system or detector, which needs to be tested with two identical detectors; (3) the LYSO crystals with the size of 6 × 6 × 25 mm^3^ are not often used in clinical PET systems. When the proposed multi-ended readout and the time–walk correction are applied to 3 × 3 × 20 mm^3^ crystals, which are usually used to build the human-body PET system, the improvement of time performance is unknown, but it is worth expecting.

The setting of trigger thresholds is important for time–walk correction, and the optimal trigger threshold is different for detectors with different structures. The trigger threshold was set to 50 mV for all tested detectors in the experiments, which is somewhat limited. The lower the threshold is, the better the time resolution of the detector is, but there will be more noise triggering, causing some invalid events. Therefore, the trigger threshold of 50 mV was selected according to the performance of the electronic system. In future work, we will optimize the electronic system and try different trigger thresholds.

The performance of time–walk correction varies with the size of the energy window. The narrow energy window is suitable for the traditional single-ended readout detector with an excellent energy resolution. However, the signal walk dramatically affects the timing measurements of a detector in the following situations: (1) When using monolithic crystals, a large energy window is required because the scintillation photons are distributed among multiple light sensors and (2) when a scintillator with low photo fraction rate (for example, LaBr3) is used, a wider energy window is usually preferred to increase the sensitivity of the detector.

In summary, in terms of the conducted simulations and experiments, the following conclusions can be drawn. Promptly improving the detector’s light output and photon transmission efficiency, along with the scintillating photons reaching the SiPM possible, can improve the detector’s timing resolution: (1) The photon transmission models of the four detectors were built with Tracepro, and, compared with the single-ended readout detector, the multi-ended detectors captured more scintillating photons over a certain time. Therefore, the light collection efficiency of the detector may be greatly increased, and the trigger time may be obtained earlier; (2) three multi-ended readout detectors were designed and tested, where the timing performance of the triple-ended readout was detected to be better than that of the dual-ended readout detector and the two-ended readout detector on the side. The more SiPMs were used, the better the timing performance was. However, the rise of the number of channels increases the complexity and cost of the PET system. The effects of time compensation are closely related to the energy resolution of the detector. The worse the energy resolution was, the better the compensation effects were; (3) based on the relationship between time and energy, a fourth-order polynomial fitting compensation algorithm was proposed. The results of the time compensation are correlated with the energy resolution of the detector.

## Figures and Tables

**Table 1:** Parameters in simulation.

Materials	Refractive Index	Absorption Rate	Thickness/Size (mm)
LYSO scintillator	1.82	0.042/mm	6 × 6 × 25
SIPM glass	1.53	0.042/mm	6 × 6 × 0.35
Diffuse reflection material	/	1.5%	0.2
Optical glue	1.53	0.08/mm	0.1

**Table 2:** Energy resolutions and energy windows.

		Dual-Ended	Side Dual-Ended	Triple-Ended
ER	Ref. detector	8.9%	9%	8.4%
	Test detector	11.8%	12.9%	11.9%
EW	Ref. detector	[440 550]		
	SiPM A	[190 310]	[180 340]	[140 200]
	SiPM B	[210 310]	[150 310]	[150 220]
	SiPM C	/	/	[150 210]

**Table 3:** FWHM CTRs of multi-ended readout detector.

		SiPM 1	SiPM 2	SiPM 3	All SiPM
Dual-ended	$CTR_{m}$	288.4	325.2	/	240.7
	$CTR_{c}$	251.9	287.1	/	**216.9**
	Ratio	12.6%	11.7%	/	9.9%
Side dual-ended	$CTR_{m}$	392.3	417.1	/	241.1
	$CTR_{c}$	293.4	316.0	/	**231.0**
	Ratio	**25.2%**	24.2%	/	4.2%
Triple-ended readout	$CTR_{m}$	294.0	364.9	315.6	224.1
	$CTR_{c}$	276.2	333.7	265.3	**203.6**
	Ratio	6.0%	8.6%	15.9%	9.1%
